# Pneumococcal Bacteremia and Cryptococcal Meningitis Dual Infection in a Patient With Multiple Myeloma

**DOI:** 10.7759/cureus.15089

**Published:** 2021-05-18

**Authors:** Abdul Raheem, Balram Rathish, Deepak Charles, Arun Wilson, Anup Warrier

**Affiliations:** 1 Internal Medicine, Aster Medcity, Kochi, IND; 2 Infectious Diseases, Aster Medcity, Kochi, IND; 3 Hematology, Aster Medcity, Kochi, IND

**Keywords:** pneumococcal bacteremia, cryptococcal meningitis, multiple myeloma, streptococcus pneumoniae, cryptococcus neoformans

## Abstract

Infections remain one of the major complications in patients with multiple myeloma, having a significant impact on morbidity and mortality. The increased risk of infection in these patients are a result of various factors contributing to the impairment of immune system caused by the disease and the chemotherapy regimens given during the treatment phases. Here we report a rare case of pneumococcal bacteraemia and cryptococcal meningitis dual infection in a patient with underlying multiple myeloma who had a favourable clinical outcome. This case also serves to highlight the importance of adult vaccinations especially in patients with underlying comorbidities.

## Introduction

Multiple myeloma (MM) forms about 10% of all hematological malignancies. The uncontrollable proliferation of plasma cells affects the normal functioning of the immune system, predisposing to infections in these patients [1]. Over the years, the survival rates in MM have improved significantly by virtue of newer treatments regimens [2-4]. Hence, the management of infections, which is one of the most common complications of MM, holds great importance [5]. A report of more than 3,000 MM patients showed that early deaths, which happened within six months of a diagnosis of MM, were directly due to infections (45%) [6]. Other reports have suggested that some of the newer chemotherapy regimens have made the patients more prone to infection and that this can lead to an increased mortality [7,8].

Bacterial as well as fungal respiratory tract infections (RTIs) are very common in patients with hematological malignancies and this may even progress to sepsis [9,10]. Opportunistic fungal infections, including cryptococcosis, generally affect immunocompromised patients due to the suppression of cell-mediated immunity [11]. Here we report a rare case of pneumococcal bacteraemia and cryptococcal meningitis dual infection in a patient with underlying MM, which proved to be a diagnostic as well as therapeutic challenge.

## Case presentation

A 60-year-old man with underlying MM and liver cirrhosis was admitted with fever and a non-productive cough for two days. He had undergone four cycles of chemotherapy with cyclophosphamide, bortezomib, and dexamethasone in the past, which were later changed to pomalidomide, dexamethasone, and zoledronate. His last chemotherapy cycle was two weeks prior to the present admission. Prior to the initiation of chemotherapy, he was advised pneumococcal, meningococcal, and other adult vaccinations but he had refused.

Clinical examination showed pallor and icterus. He had tachycardia, tachypnea, and an elevated temperature of 100 F. Examination of the chest revealed diffuse crepitations on bilateral lung fields. An abdominal exam revealed hepatomegaly with ascites. Neurological examination was unremarkable with no signs of meningism.

**Figure 1:**
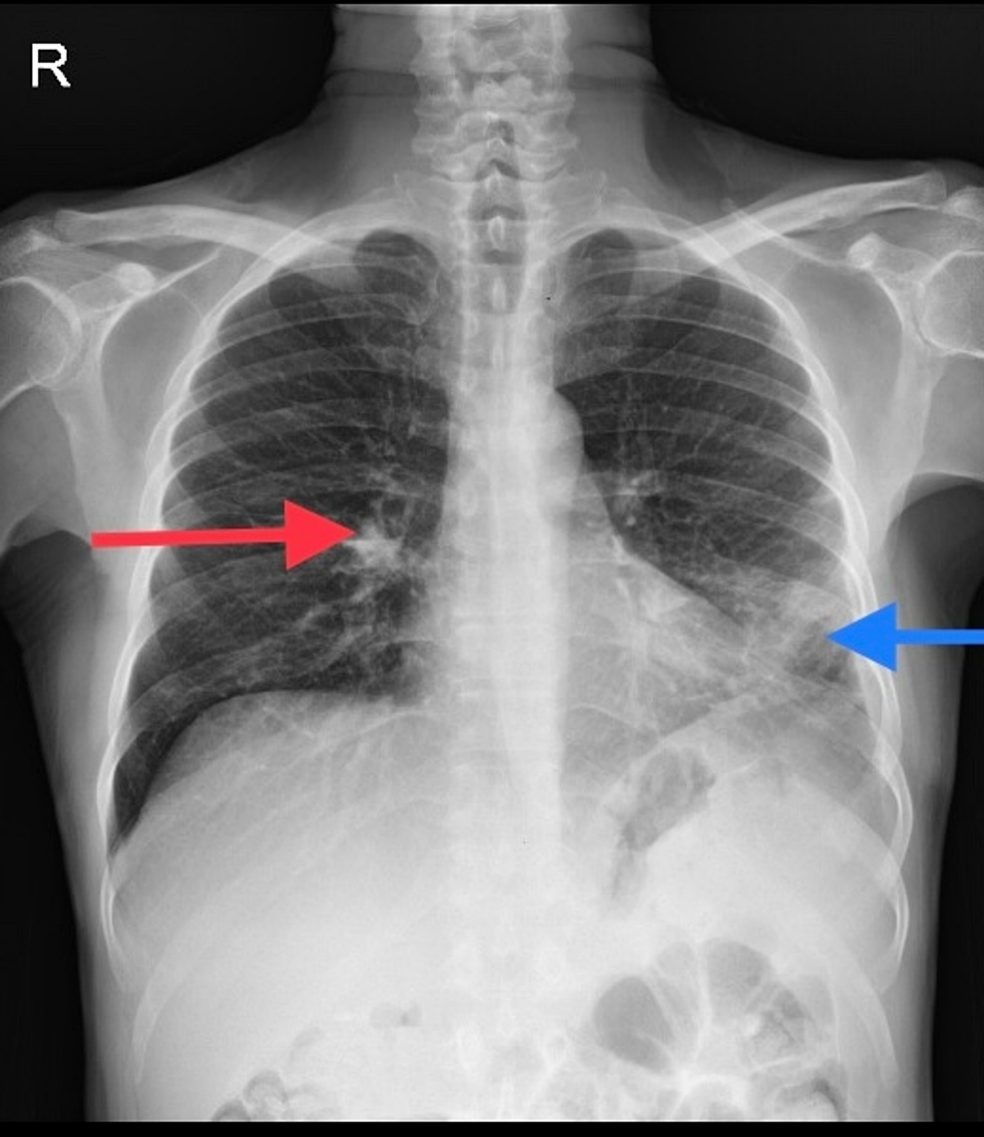
Chest X-ray showing right middle lobe (red arrow) and left lingular consolidation (blue arrow).

**Figure 2:**
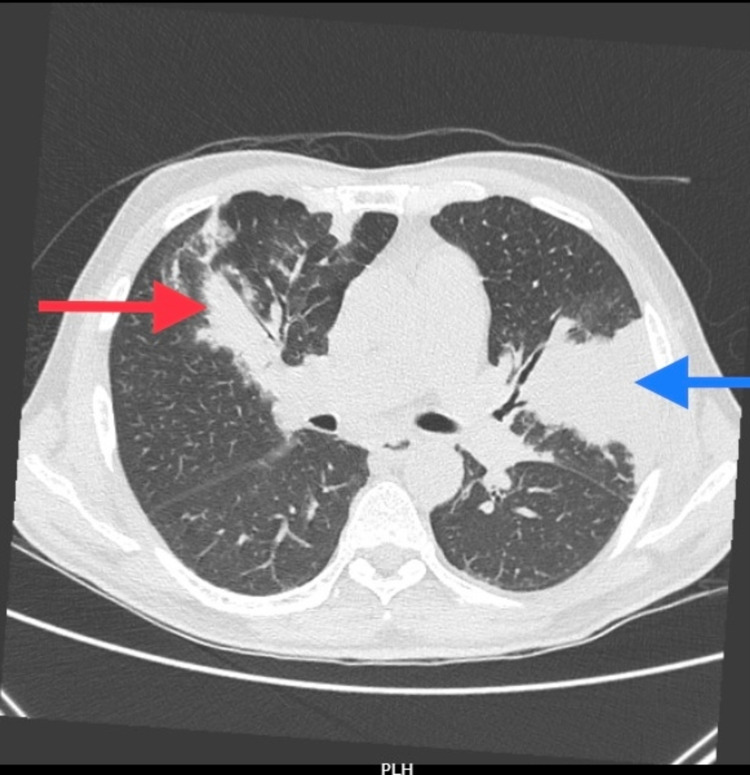
CT chest showing a consolidation involving the left lingular segment (blue arrow) along with peri-bronchial consolidation in the anterior segment of the right upper lobe (red arrow). CT - computed tomography

A chest X-ray showed the right middle lobe and left lingular consolidation (Figure 1). His C-reactive protein was 172 (<10 mg/dL), serum sodium was 125 (134-145 mmol/L), and albumin was 1.9 (3.5-5.5 g/dL). A computed tomography (CT) chest showed consolidation with collapse involving the left lingular segment, along with peri-bronchial consolidation in the anterior segment of the right upper lobe suggestive of an infective etiology (Figure 2). A diagnosis of community-acquired pneumonia was made and he was started on broad-spectrum antibiotics (ceftriaxone) as per the local antibiogram after obtaining blood, sputum, and urine for microbiological culturing.

On day 2, his blood culture grew gram-positive cocci, which were identified to be penicillin-sensitive *Streptococcus pneumoniae*. His antibiotic was continued and he remained afebrile. However, on day 4, he developed a persistent fever. The ceftriaxone was changed to pipercillin-tazobactum. Repeat blood cultures on day 7 were negative with no persistent bacteremia or secondary infection, and CRP showed a reducing trend. But he continued to have febrile episodes despite completing 10 days of antibiotics. In view of persistent fever in an immunosuppressed patient, possibilities of local complications like empyema and lung abscess were considered and levofloxacin was added. A repeat CT chest along with CT abdomen was performed, which did not show any local collections in the lung or any visceral abscess. Ascitic fluid analysis revealed no features of spontaneous bacterial peritonitis. An echocardiogram revealed no evidence of Infective endocarditis.

**Figure 3:**
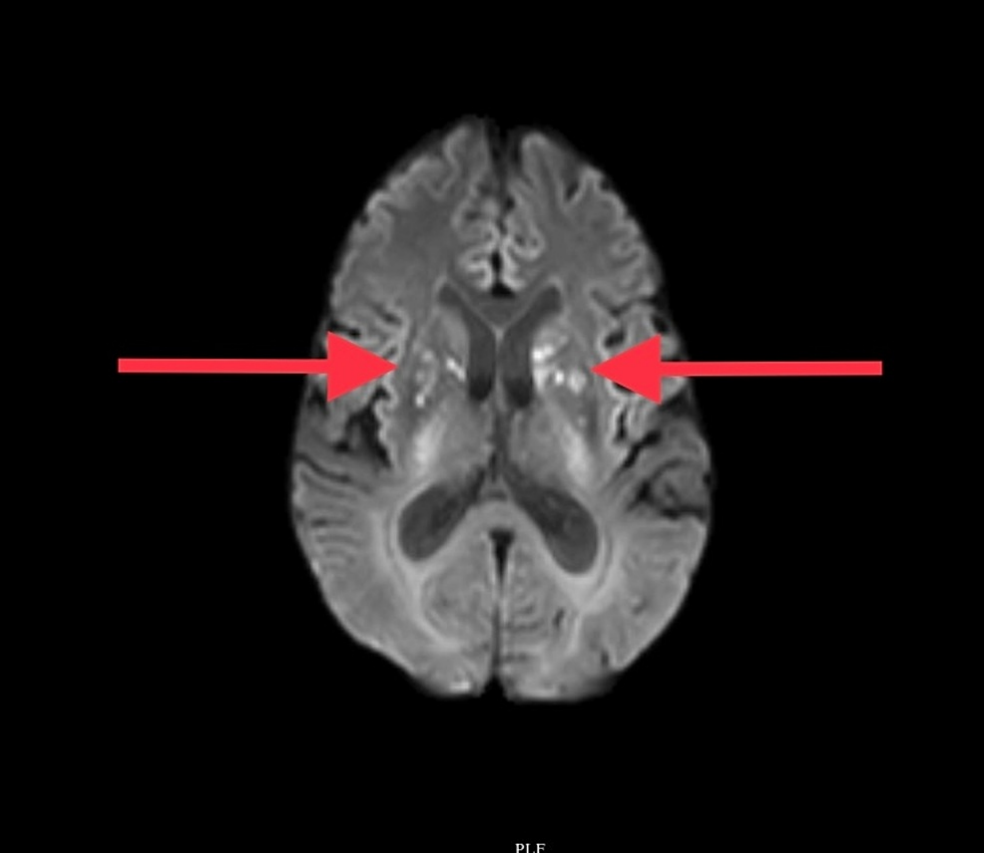
Diffusion-weighted MR showing ischemic infarcts in bilateral internal capsules. MR - magnetic resonance

On day 12, he developed altered sensorium with slurring of speech. MRI brain revealed ischemic infarcts in the internal capsules (Figure 3). Since fever was persisting, the possibility of a meningeal infection was suspected even though there were no clinical signs of meningism. Since pipercillin-tazobactum and levofloxacin would not penetrate the blood-brain barrier adequately, the antibiotic was changed to ceftriaxone at a meningitic dose. Serum galactomannan was elevated at two (<0.5), and hence voriconazole was added. Cerebrospinal fluid (CSF) study showed a total count of 45 cells/µL (0-5 cells/µL), which were all lymphocytes, sugar of 110 mg/dL (paired serum sugar of 82 mg/dL), and protein of 135 (15-60 mg/dL). Tubercular and fungal meningitis were considered the differential diagnosis. Acid-fast staining and GeneXpert® (Cepheid, Sunnyvale, California, United States) were negative. CSF India ink staining revealed capsulated yeasts suggestive of *Cryptococcus neoformans,* which was confirmed using a lateral flow assay for cryptococcal antigen. He was treated with flucytosine and amphotericin-B for two weeks following which he improved clinically. He remained afebrile, conscious, and alert throughout the rest of the hospital stay and was discharged at the end of two weeks with oral fluconazole to be continued for 10 weeks. A repeat CSF study done on follow up was negative for *Cryptococcus*.

## Discussion

MM patients tend to have a low immune response to vaccines, which predisposed them to contract the infection despite vaccinations [12,13]. This patient was advised to take pneumococcal vaccination prior to the initiation of chemotherapy, but he had refused which directly contributed to pneumococcal pneumonia and bacteremia. Assessment for the risk of infection in MM patients should be done before and during the treatment of MM. Detailed past medical history, risk factors, screening for any infections, and the vaccinations administrated should be taken to make relevant prophylactic measures for each patient. It is recommended to vaccinate against *S. pneumoniae* at the time of diagnosis of MM in the patient and vaccination against influenza for family members yearly to prevent infections to some extent. It is better to recommend antibacterial, antifungal, and antiviral prophylaxis considering the phase of the disease and treatment type for each patient [14].

Cryptococcal infection in the central nervous system may manifest as meningitis and as meningoencephalitis with a variable clinical presentation. *Cryptococcus* causes opportunistic infection resulting in high morbidity and mortality. Some reports have shown that the acute mortality due to cryptococcal meningitis remains high (30% to 50%), even with adequate antifungal therapy. Other studies have shown that cryptococcal meningitis can cause considerable morbidity and survivors may have a residual visual impairment, hearing loss, or neurocognitive impairment that may be irreversible [15,16]. Our patient however had a very good clinical outcome with no residual neurological impairment. He was treated with amphotericin B and flucytosine. Combination therapy using amphotericin B and flucytosine had shown increased rates of fungal clearance when compared with four-week amphotericin monotherapy. The currently accepted treatment regimen for cryptococcal meningitis involves induction with amphotericin B (0.7-1.0 mg/kg/day) along with flucytosine (100 mg/kg/day) for two weeks. Followed by consolidation and maintenance, fluconazole at 400 mg/day for 8 to 12 weeks and at 200 mg/day for 6 to 12 months, respectively [17,18].

The patient was having hyponatremia on admission and the initially altered sensorium was thought to be due to that, but could very well have been due to the acute cerebral infarcts. The sensorium remained the same even after the correction of serum sodium and the fever persisted even after pneumonia subsided. This led to the search for another underlying infection and identification of cryptococcal meningitis. Although multiple infections may be encountered in immunosuppressed patients, this type of dual infection involving an unusual pathogen causing a bloodstream infection with concurrent fungal meningitis is very rare and the timely diagnosis together with rapid institution of appropriate therapy resulted in a favourable outcome in our patient.

## Conclusions

Dual infections are rarely encountered especially in the setting of immunosuppression. This case serves to highlight that infections in immunocompromised patients should be high on the differential in patients presenting with symptoms in order to prevent morbidity and mortality. If the symptoms do not subside even with appropriate treatment of the infection, co-existing infections should be high on the list of differentials in such patients.

Timely diagnosis and rapid institution of appropriate therapy are paramount in improving clinical outcomes in such patients. Adult vaccination also forms an important step in the treatment of an immunocompromised patient in order to prevent vaccine-preventable diseases. Patients should be encouraged to get vaccinated at the earliest. Patients should also be educated regarding the lifestyle modifications necessary to minimize the risk of acquiring common infections.
